# Stress-Induced Hyperglycemia: Consequences and Management

**DOI:** 10.7759/cureus.26714

**Published:** 2022-07-10

**Authors:** Deepanjali Vedantam, Devyani S Poman, Lakshya Motwani, Nailah Asif, Apurva Patel, Krishna Kishore Anne

**Affiliations:** 1 Internal Medicine, Kamineni Academy of Medical Sciences and Research Centre, Hyderabad, IND; 2 Research, Smolensk State Medical University, Smolensk, RUS; 3 Research and Development, Smt. NHL (Nathiba Hargovandas Lakhmichand) Municipal Medical College, Ahmedabad, IND; 4 Research, RAK (Ras Al Khaimah) College of Medical Sciences, Ras Al Khaimah, ARE; 5 Research, GMERS (Gujarat Medical Education & Research Society) Gotri Medical College, Vadodara, IND; 6 Internal Medicine, National Pirogov Memorial Medical University, Vinnytsya, UKR

**Keywords:** insulin in icu, admission hyperglycemia, in-hospital mortality, continuous glucose monitoring systems, nutrition in critical care, insulin protocol, diabetes and hospitalar hyperglycemia, de novo diabetes mellitus, critically ill patients, stress hyperglycemia

## Abstract

Hyperglycemia during stress is a common occurrence seen in patients admitted to the hospital. It is defined as a blood glucose level above 180mg/dl in patients without pre-existing diabetes. Stress-induced hyperglycemia (SIH) occurs due to an illness that leads to insulin resistance and decreased insulin secretion. Such a mechanism causes elevated blood glucose and produces a complex state to manage with external insulin. This article compiles various studies to explain the development and consequences of SIH in the critically ill that ultimately lead to an increase in mortality while also discussing the dire impact of SIH on certain acute illnesses like myocardial infarction and ischemic stroke. It also evaluates multiple studies to understand the management of SIH with insulin and proper nutritional therapy in the hospitalized patients admitted to the Intensive care unit (ICU) alongside the non-critical care unit. While emphasizing the diverse effects of improper control of SIH in the hospital, this article elucidates and discusses the importance of formulating a discharge plan due to an increased risk of type 2 diabetes in the recovered.

## Introduction and background

Hyperglycemia in critically ill patients is a commonly observed finding usually evident in the first 48 hours on admission to the ICU in at least 50% of the patients [1]. Numerous studies have revealed a significant relationship between blood glucose concentrations on admission to ICU or in ICU and the criticality of the outcome [2,3]. Stress-induced hyperglycemia (SIH) brings up a state of insulin resistance and increased blood glucose through several mechanisms [4]. Counterregulatory hormones like catecholamines, cortisol, glucagon, and growth hormone disturb glucose hemostasis. Also, an increase in the inflammatory cytokines further worsens the metabolic milieu [4]. Therefore, hepatic gluconeogenesis is not under control. Glucose uptake by the skeletal muscle via the glucose transporter type 4 (GLUT-4) is also impaired [4]. Furthermore, insulin levels themselves are low to combat the state of hyperglycemia [5]. Hyperglycemia, defined as blood glucose levels >180mg/dl, demonstrated an increase in mortality in a retrospective study performed in the USA, emphasizing the importance of strict glycemic control [6]. Moreover, according to studies, it is not diabetic hyperglycemia (DH) but the state of SIH that is mainly responsible for increased mortality and morbidity [7]. A coordinated approach from a multidisciplinary team is required to safely achieve proper glycemic control in the inpatient setting [8]. A process including an adequate diagnosis, optimal management in the ICU settings, and resolute continuity of care significantly helps reduce morbidity risks [9]. Intravenous insulin therapy has been a standard of care for hyperglycemia [10]. Continuous glucose monitoring (CGM) systems have recently come into play at hospitals. They are devices that allow continuous glucose monitoring without much exposure to healthcare professionals [10,11]. Despite not having any striking evidence of improved patient outcomes, it has considerably reduced the risk of hypoglycaemic episodes [12,13]. An increase in blood glucose variability and the number of hypoglycaemic events are further recognized to lead to poor outcomes [14,15]. This review aims to outline the management of SIH by discussing in detail the investigations deemed necessary, insulin requirements, and nutritional advancements while highlighting the possible complications.

## Review

**Figure 1:**
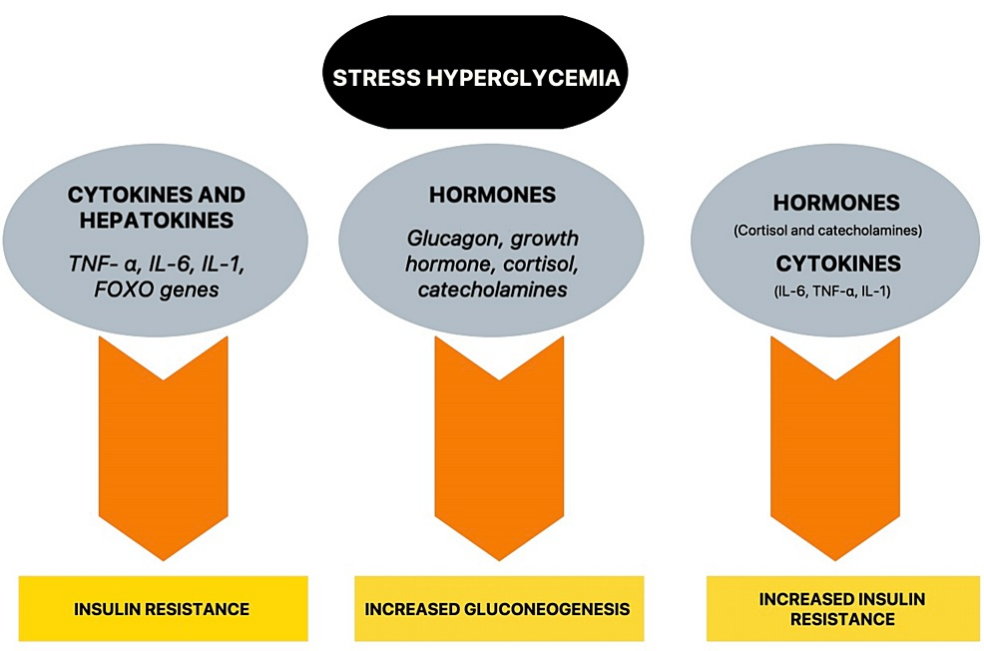
Pathophysiology of Stress Hyperglycemia TNF-α: Tumor necrosis factor-α; IL-6: Interleukin-6; IL-1: Interleukin-1; FOXO: Forkhead Box O Image credit: Deepanjali Vedantam

Counter-regulatory hormones and pro-inflammatory cytokines are responsible for the metabolic milieu that develops in SIH [1]. Increased gluconeogenesis and insulin resistance are important factors [16]. Interleukin-1 (IL-1), interleukin-6 (IL-6), and tumor necrosis factor-α (TNF-α) are inflammatory cytokines that cause insulin resistance and also suppress insulin release, an effect that is concentration-dependent [17]. Increased levels of IL-6 in the serum are associated with insulin resistance [18], which promotes hyperglycemia by releasing glucose from hepatic glycogen reserves [19]. Additionally, hyperglycemia raises IL-6 levels in the blood, presumably due to elevated production in monocytes [20]. TNF-α is associated with the severity of sepsis and is the primary mediator in the development of sepsis [21, 22]. TNF-α causes insulin resistance in animals by itself [23] or by increasing circulating levels of free fatty acids [24, 25]. The hypothalamic-pituitary-adrenal (HPA) axis is activated in hyperglycemia-induced by stress, leading to increased cortisol secretion from the adrenal gland. Cortisol synthesis is necessary for maintaining cellular hemostasis and organ systems [26]. Cortisol, catecholamines, glucagon, and growth hormone are all counter-regulatory hormones that decrease insulin release by enhancing the activity of pancreatic alpha cells [16]. Increased hepatic gluconeogenesis is regulated by catecholamines and cytokines, whereas glucagon is a primary mediator [27]. Catecholamines additionally limit insulin binding, insulin activation by suppressing tyrosine kinase activity, and glucose uptake in the periphery by GLUT-4 [28, 29]. Similarly, Glucocorticoids also restrict glucose uptake in the peripheral tissues, while growth hormone prevents insulin activation on tyrosine residues [30-32]. Therapeutic interventions like nutrition also play a vital role in developing hyperglycemia [33]. Another important mechanism involved in SIH is Forkhead Box O (FOXO) transcription factors. The deletion of FOXO transcription factors reduces SIH by modifying the gene expression. The modifications help to promote insulin resistance and glycogenolysis in the liver. It also indirectly decreases lipolysis in the adipose tissue [34]. SIH is associated with increased gene expression of hepatic glucose-6-phosphatase (G6PC), a gluconeogenic gene regulated in part by FOXO [35]. Critically ill patients have a high insulin-like growth factor-binding protein-1 (IGFBP-1), a liver-derived protein that prolongs insulin-like growth factor activity and is ordinarily inhibited by insulin [36]. The rise in IGFBP-1 in humans is associated with insulin resistance in the liver and critical illness mortality [37]. FOXO also regulates IGFBP-1, implying that the transcription factor family potentially plays a significant role in SIH [35, 38]. Reduced blood glucose levels in SIH were linked to lower levels of cytokines and FOXO-regulated hepatokines, implying that they play a vital role in developing hyperglycemia [34] (Figure 1).

Mortality and consequences of SIH

Hyperglycaemia is associated with poor outcomes and with an increased risk of mortality. A large study by Mamtani et al. demonstrated this association by taking under 739,152 non-diabetic ICU patients admitted during 2007-2016 from the Australian and New Zealand Intensive Care Society (ANZICS) Adult Patient Database (APD). They conducted the research by quantifying hyperglycemia in these patients using a midpoint blood glucose level (MBGL). Outcomes were analyzed, including the length of hospital stay (LOS) and mortality. In this extensive study of non-diabetic ICU patients, the degree of hyperglycemia defined by MBGL was significantly associated with higher mortality in-hospital and LOS. The above associations strengthened in patients admitted for a neurological disease, trauma (especially head trauma), and coma patients [39].

Additionally, Sleiman et al. studied the association between hyperglycemia and increased mortality in elderly patients without diabetes in the sub-intensive care unit. The sub-intensive care unit is the level of care between the ICU and regular wards. This study included 1,229 patients with a mean age of 79.6 +/- 8.4 years. Among these, 822 patients were non-diabetic, whereas 333 patients had diabetes. Age, sex, acute physiology score, mental and functional status, presence of comorbidities, serum albumin and cholesterol, fasting serum glucose, and LOS were considered. In-hospital mortality was the primary outcome, and 45-day mortality was the secondary. Newly diagnosed hyperglycemia with values above 181 mg/dl was associated with higher mortality risk in the hospital. On the other hand, higher 45-day mortality was observed in patients with newly diagnosed serum blood glucose above 127 mg/dl compared to previously diagnosed diabetics. Therefore, concluding the risk of higher mortality rate among elderly patients with In-hospital hyperglycemia [40]. 

Another study by Godinjak et al. concluded that SIH is associated with elevated mortality risk and poorer outcomes than DH in the ICU. The predictors of poor outcomes were mentioned as well. This study included 100 patients in the ICU for one year. It considered the age, gender, daily blood glucose, highest blood glucose values, overall glycemic variability, usage of vasopressors and corticosteroids, mechanical ventilation, and total duration of hospital stay in the ICU to establish a relationship between the blood glucose and outcome in the critically ill. Adverse sequelae were strongly associated with increased glycemic variability; hence, revealing it to be the strongest predictor. SIH also increased the incidence of critical illness polyneuropathy (CIP) [41]. Rau et al. conducted a study to elucidate higher mortality among trauma patients with SIH rather than DH. They retrieved hospitalized patients between January 2009 to December 2015 from the Level 1 trauma center. The study considered patients without diabetes with blood glucose levels >200mg/dl to be under SIH, while previously known diabetic patients with blood glucose above 200 mg/dl were considered to be under DH. SIH was associated with a higher injury severity score. While the characteristics and severity of an injury correlate with higher mortality among patients with SIH, the study also revealed that the effect is owed to the different pathophysiological mechanisms of SIH compared to DH. Therefore, SIH had significantly demonstrated higher mortality when compared to DH when controlling for age, sex, pre-existing comorbidities, and injury severity score [42] (Table 1).

**Table 1:** Summary of Studies Explaining the Link Between Stress Hyperglycemia and Elevated Risk of Mortality SIH: Stress-induced hyperglycemia; ICU: Intensive care unit; DH: Diabetic hyperglycemia; DM: Diabetes mellitus; SHR: stress hyperglycemic ratio; HbA1c: hemoglobin A1c; LOS: length of hospital stay; MBGL: midpoint blood glucose level

Reference	Year	Population	Methods	Inclusion and exclusion criteria	Outcomes	Comments
Mamtani et al. [39]	2019	739,152 from the Australian and New Zealand Intensive Care Society (ANZICS) Adult Patient Database (APD)	Association of outcomes like LOS and in-hospital mortality tested using multivariable, mixed effects, 2-level hierarchical regression from the ANZICS which is the largest dataset with over 2 million ICU registered admissions.	Included 739,152 ICU patients without pre-existing diabetes and available following data: lowest and highest blood glucose level within the first 24 hours of admission, LOS, hospital death, predicted risk of death and ICU admission diagnostic code (derived from ANZICS modification of APACHE -III scoring system)	Degree of hyperglycemia was quantified using MBGL. The fourth, third and second MBGL (compared to the first) quartiles were associated with hospital mortality (odds ratio 1.34, 1.05 and 0.97, respectively) and longer hospital stay (1.56, 1.38 and 0.93 days, respectively).	Hyperglycemia in non-diabetic critically ill patients was associated with LOS and higher in-hospital mortality, especially in patients with trauma, neurological disease and coma patients.
Sleiman et al. [40]	2008	1229 patients admitted to the Sub-ICU from 2003-2006	Retrospective cohort study on 1229 Sub-ICU patients where variables including age, sex, mental and functional status, Acute physiology score, comorbid conditions, serum albumin, serum cholesterol, fasting serum glucose, and length of stay where taken into account.	822 patients without a history of DM and 333 patients with a prior history of DM were selected. Patients with AMI and extreme sting blood glucose values (<60 and >500 mg/dl) were excluded.	Primary outcome was in-hospital mortality and secondary outcome was 45 day mortality. Newly recognized hyperglycemia (>181 mg/dl) was associated with high in-hospital mortality (adjusted odds ratio=2.7, 95% confidence interval=1.6-4.8) and higher 45-day mortality.	Increase in in-hospital and 45-day mortality is linked to new onset hyperglycemia in the hospital.
Godinjak et al. [41]	2015	100 medical ICU patients	Patients were divided into normoglycemic, SIH, and DM. Retrospective and prospective observational study where the simplified acute physiology score was calculated 24 hours after admission which correlates with mortality rate.	Patients admitted to the ICU and studied were grouped into five categories: Respiratory (43%), cardiovascular (17%), septic shock (15%), neurological (15%), other causes (10%)	A significant difference in maximum blood glucose level was noted in patients with adverse outcomes (p= 0.0277) and patients with DM under continuous insulin infusion and normoglycemia did not have any difference in complications while patients with SIH had a severe prognosis.	Poorer outcomes with SIH when compared to DH. Greater glycemic variability is associated with adverse outcomes and is a predictor of poor prognosis.
Rau et al. [42]	2017	Adult hospitalised trauma patients from 2009-2015 retrieved from the Trauma Registry System at a level 1 trauma centre	Hba1c >6.5% and history was used to diagnose patients with DM. DH and SIH was diagnosed by blood glucose >200mg/dl in diabetics and non-diabetics, respectively. Logistic regression was used to analyse the outcomes in patients with DH and SIH.	Adult patients with t >20 years and available data on serum glucose, Hba1c and history of DM were included in the study. Patients with inadequate data were excluded.	SIH had 2.4-fold higher odds of mortality (95% CI 1.46–4.04; p = 0.001) than DH.	Higher mortality and injury severity score among patients with SIH when compared to DH.

It's worth noting that many critical illnesses strongly associate SIH with adverse outcomes such as increased mortality. Acute myocardial infarction (AMI) is one such illness in which SIH is common. Adverse events in patients with myocardial infarction (MI) increase when associated with SIH, irrespective of their previous diabetic status or glycemic control. One such study by Rajpurohit et al. demonstrated this occurrence by evaluating 100 patients above 18 years admitted to the ICU with acute MI. Out of the 100 patients, 50 patients admitted to the ICU with blood glucose above 180mg/dl were grouped under SIH, and the rest of the 50 patients were classified as normoglycemic. The study showed an elevated incidence of complications such as arrhythmias, cardiogenic shock, progression to severe heart failure, and a significant increase in mortality. Hence, SIH in patients with MI had a critical role in the outcome [43]. Chen et al. studied the sequelae in elderly patients with AMI with SIH by determining the association between stress hyperglycemic ratio (SHR) and in-hospital outcomes in elderly patients with AMI. SHR is the ratio of the fasting glucose concentration at admission and the HbA1c. The outcome and increased fatality rate in a patient with an acute illness can be predicted using an SHR. It is an index of relative stress hyperglycemia and is used as a prognostic value in AMI. In this, 341 patients diagnosed with AMI over 75 years were analyzed to identify a link between SHR and in-hospital outcomes. All-cause mortality and adverse cardiac and cerebrovascular events such as cardiogenic shock, reinfarction, mechanical complications of MI, stroke, and severe bleeding comprised the in-hospital outcomes. This study found that SIH is an independent predictor of poor outcomes in elderly patients with AMI [44]. Along similar lines, another study by Khalfallah et al. conducted on 660 patients with ST-elevation MI managed with percutaneous coronary intervention elucidated the adverse outcomes of SIH. Patients were studied based on the presence or absence of SIH. On evaluation, the evidence suggested an elevated incidence of the no-reflow phenomenon, contrast-induced nephropathy, cardiogenic shock, and higher mortality in these patients [45].

 Another noteworthy critical illness associated with SIH is acute cerebrovascular accident. As MI, acute illness such as stroke is related to poor outcomes with elevated blood glucose even without pre-existing diabetes. SIH in patients with ischemic stroke is associated with an increased risk of recurrence and all-cause mortality. Data from the Abnormal Glucose Regulation in Patients with Acute Stroke across China (ACROSS-China) registry had demonstrated that increased SHR was associated with increased recurrence and adverse outcomes leading to mortality in patients with acute ischemic stroke [46]. Hemorrhagic transformation of acute ischemic stroke is one such complication known consequence of SIH. Yuan et al. demonstrated this association by using the SHR. It had revealed that hemorrhagic transformation had frequently occurred with ischemic stroke [47].

Similarly, Li et al. conducted a study on 8622 patients from the China National Stroke Registry II in 2020. SHR was estimated, and patients were analyzed for a year for severe neurological deficit and all-cause death. After one year, 1189 patients had a severe neurological deficit and 678 patients died, showing a significant association between SHR and severe neurological deficit. There was a definite increase in mortality within a year of an episode of acute ischemic stroke [48].

Incidence of type 2 diabetes in patients recovered from SIH

We now know that SIH is associated with an elevated risk of poor acute outcomes, but also it increases the risk of prolonged illnesses such as type 2 diabetes. SIH has increased the risk of type 2 diabetes after recovery. A few studies to validate the association have been considered (Table 2).

**Table 2:** Summary of Studies Explaining the Association Between Stress Hyperglycemia and Incidence of Type 2 Diabetes ICU: Intensive care unit; SIH: Stress-induced hyperglycemia; MI: Myocardial infarction; HbA1c: Hemoglobin A1c; AMI: Acute myocardial infarction; CI: Confidence interval; HR: Hazards ratio

Reference	Year	Population	Design	Methods/Results	Comments
Plummer et al. [49]	2016	17,074 adults above 18 years in the ICU	Retrospective cohort	Blood glucose above 200mg/dl measures within 24 hours in ICU were followed for 30 days after discharge. Patients with SIH had roughly twice the probability of developing diabetes as those without it (HR 1.91 (95% CI 1.62, 2.26), p<0.001).	SIH is associated with a subsequent risk of type 2 diabetes
Moradi et al. [50]	2015	98 patients in the emergency department at Firouzgar Hospital	Cross sectional study	Blood sugar levels above 180mg/dl and no history of diabetes were enrolled. HbA1c above 6.5% were excluded form the study. Screening for diabetes was performed after three months. 25.8 % developed pre-diabetes, statistically significant relationship between diabetes and gender (P=0.027)	Statistically significant association between SIH and risk of type 2 diabetes, males affected more than females
Hsu et al. [51]	2015	9528 critically ill patients studied from the Taiwan National Health Insurance Research Database	Cohort study	Patients with critical illness like sepsis, stroke, MI and septic shock vs non critically ill. statistically significant risk is noticed in patients in the critical illness cohort (adjusted hazard ratio, aHR = 1.32; 95% confidence interval, CI 1.16-1.50). Higher risk in those with sepsis or septic shock (aHR = 1.51, 95% CI 1.37-1.67), followed by AMI.	Certain critical illnesses are associated with a high risk of developing type 2 diabetes
Kar et al. [52]	2019	40 patients from tertiary, mixed medical-surgical ICU	Cohort study	Patients admitted to medical-surgical ICU and survived until hospital discharge were eligible. HbA1c and oral glucose tolerance test was measured three months and 12 months after discharge. Mean HbaA1c increased from baseline during the study: -1.2 to 2.5 mmol/mol at three months and 0.98-5.59 mmol/mol at 12 months (p = 0.02).	Survivors experience diabetes and pre diabetes after a critical illness

Plummer et al. studied 17,074 adult patients over 18 years in a retrospective cohort study in South Australia. The included patients were studied for eight years after surviving in an ICU. Patients without pre-existing diabetes had a 4.8% chance of developing type 2 diabetes after a critical illness. As a result, independent of age or degree of sickness, the risk doubled in individuals who have recovered from critical illness [49]. In a similar study, Moradi et al. analyzed 98 patients at Firouzgar Hospital's emergency department over two years, from 2012 to 2014. Patients included in the study were those with head trauma, sepsis, stroke, abdominal surgery, trauma, MI, and subarachnoid hemorrhage. The trial comprised patients with blood sugar levels > 180 mg/dl on admission and no previous history of diabetes. A fasting blood sugar level above 126 mg/dl or blood sugar over 200 mg/dl after two hours of a glucose load was considered a diabetic state. After three months of follow-up, the relationship between SIH and diabetes was found to be statistically significant. Moreover, there was a more positive correlation between gender and future diabetes risk in men than in women. The mean HbA1C three months after admission had no statistically significant relationship with the background incident. According to the study, scientific evidence linking BMI to the development of diabetes has not been found [50]. A cohort study with data from the Taiwan National Health Insurance database studied 9528 patients with critical illnesses such as septicemia, septic shock, AMI, and stroke. The control group comprised 9528 healthy patients. The critically ill group had a significantly increased risk of developing type 2 diabetes. Furthermore, it showed that individuals with sepsis or septic shock had a higher chance than those with other acute conditions. As a result, certain critical illnesses are at an increased risk of type 2 diabetes [51]. The findings of the above studies are compared along the lines of another cohort study by Kar et al. that includes patients who acquired SIH during admission and survived until discharge. HbA1c was recorded on entry to the ICU. The eligible patients returned for HbA1c testing and an oral glucose tolerance test three months and 12 months later. Gastric emptying was also studied using an isotope breath test. While considering an increase in HbA1c from baseline during the study, the research indicates a significant risk of diabetes and prediabetes in critical illness survivors. Furthermore, there was no substantial difference in gastric emptying [52].

Management of SIH

It is well established that optimal blood glucose control is critical in improving clinical outcomes in a patient. Several studies have proposed appropriate management of SIH; however, the agreement on a glycemic goal is still controversial. Many healthcare providers do not have a proper formulated approach to managing SIH. A study showed that every 10mg/dl increase in the blood glucose level above 120mg/dl was associated with an exponential rise in patient mortality [53]. A large cohort study proved that glycemic variability had adverse outcomes and increased mortality [54]. Rapid blood-glucose variations in critically ill patients under tight glucose control necessitate quick recognition and frequent, accurate, and timely glucose measurement for the optimal insulin infusion administration, which is a time-consuming process [55]. The measurement method (central laboratory, arterial blood gas machine, or point-of-care (POC) glucometers) and type of sample (whole blood vs. plasma) affect blood results [56-58]. Blood glucose levels in the ICU are measured using POC glucometers. These enable rapid and effective glucose measurement as it would in the venous sample in the critically ill [59]. The ability to quickly measure blood glucose levels with minimal blood volumes is a significant benefit of POC glucose monitoring devices. It is most accurate when used within the normal glucose range, which could be a concern with strict glycemic control as detecting high and low blood glucose levels is required. The mechanism used by a POC meter (glucose oxidase vs. glucose-1-dehydrogenase) impacts accuracy and the likelihood of interference from patient physiology, other circulating substances, and sample source [60]. It is vital that blood glucose levels be measured every two hours in unstable patients and can be taken every four hours once the patient is stabilized [61]. However, it is a time-consuming process that requires highly efficient human work. The extremes of glucose variability and manual burden may be avoided with the aid of CGMs. Their use might eliminate the impact of poor peripheral perfusion and other confounding variables that interfere with POC monitoring. It would also enable rapid recognition of fluctuations attributable to changes in insulin requirements.

Additionally, the integration of microtechnology may facilitate the development of a closed-loop system that functions as an acute beta-cell modulator or artificial pancreas [62, 63]. It would allow bedside caregivers to cut down on the typical daily time (estimated to be between 100 and 120 minutes) needed to measure glucose and adjust intensive insulin therapy (IIT). Various continuous methods for assessing, such as an indwelling arterial or venous catheter or an interstitial/ subcutaneous analysis, are being explored [64].

Protocols to administer glucose can range from simple to sophisticated algorithms. While basic algorithms are easy to follow at the bedside, it is difficult to appropriately manage patients with insulin resistance due to critical illness or in patients with pre-existing diabetes [65]. Hence, more advanced algorithms are incorporated to manage patients in the ICU. Such complex algorithms combine relevant data for specific patients, such as previous blood glucose concentrations and trends over time, present and previous insulin infusion rates, and dietary input. They help to enhance glucose control in critically ill patients [66].

Numerous organizations and associations have proposed various guidelines to control blood glucose levels in the ICU, reflecting the discrepancies in managing stress hyperglycemia. American College of Physicians guidelines in 2011 recommends liberal blood glucose levels at 7.7-11.1 mmol/l rather than intensive glycemic control at levels such as 4.4 to 6.1 mmol/l. Similarly, the American Diabetes Association (ADA) 2012 recommended a target blood glucose range of 7.7- 9.9 mmol/l [67]. Society of Critical Care Medicine (SCCM) further recommended a target range of 5.5 to 8.3 mmol/l with a maximum blood glucose of 9.9 mmol/l [68]. Although IIT can be used to achieve tight blood glucose targets, it was associated with an increase in mortality rather than a beneficial association [69]. However, strict blood glucose targets can be considered in critically ill patients with stroke or cardiac failure [70]. Patients admitted due to sepsis and undergoing treatment with hydrocortisone had no significant difference in mortality with strict blood glucose targets like 4.4 to 6.1 mmol/l and those with a target blood glucose of 8.3 mmol/l and less [68].

On the other hand, patients with severe sepsis did not benefit from strict blood glucose targets but suffered from complications such as hypoglycemia [71]. Therefore, the current guidelines recommend the maintenance of target blood glucose ranging between 7.7 to 10.0 mmol/l. Neither strict targets (4.4 to 6.1 mmol/l) nor liberal ranges (10.0 to 11.1 mmol/l) are recommended. Hence, avoiding severe hyperglycemia and the risk of iatrogenic hypoglycemia [41].

**Figure 2:**
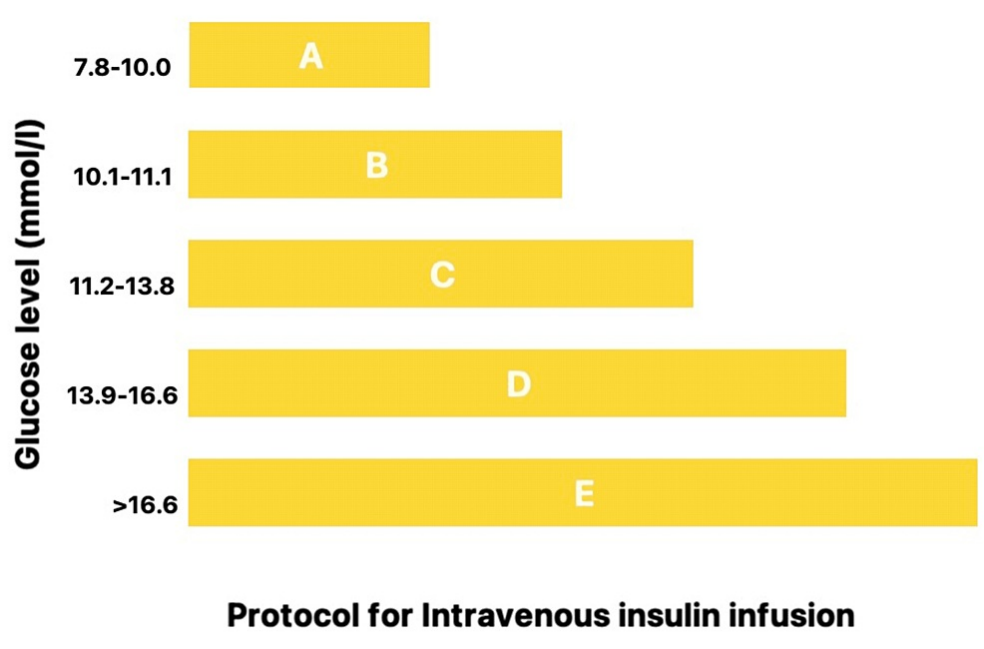
Protocol for Intravenous Insulin Infusion Image credit: Deepanjali Vedantam

Continuous intravenous insulin infusion (CIIT) is usually considered in critically ill patients in the ICU. Factually speaking, insulin has a short half-life of fewer than 15 minutes; hence, it can rapidly adjust glucose levels and reach the target range within four to eight hours [61,72]. Heidary et al. reported in their study about the effect of a loading dose of magnesium on insulin resistance in ICU patients with SIH. A loading dose of magnesium (7.5 g of magnesium sulfate in 500 mL normal saline as an intravenous infusion over eight hours) or placebo was administered to 70 patients with SIH. The study concluded that a single loading dose of a magnesium bolus had been shown to reduce insulin resistance and improve these patients' compliance [73]. The ideal treatment protocol should establish balance and stability, efficiently achieve and maintain target blood glucose levels, consider the rate of change in blood glucose levels, and lead to the lowest tendency to develop hypoglycemia. Additionally, as seen in the protocol, nurses should be able to receive and adhere to specific guidelines for titration and frequency of glucose monitoring [41] (Figure 2) (Table 3).

**Table 3:** Protocol for Intravenous Insulin infusion IV: intravenous; IU/h: International units/hour

A	Start IV insulin infusion with 1 IU/h
B	Start IV insulin infusion with 2 IU/h
C	Bolus 2 IU insulin IV and start IV insulin infusion with 2 IU/h
D	Bolus 4 IU insulin IV and start IV insulin infusion with 2 IU/h
E	Bolus 4 IU insulin and start IV insulin infusion with 4 Iu/h

**Figure 3:**
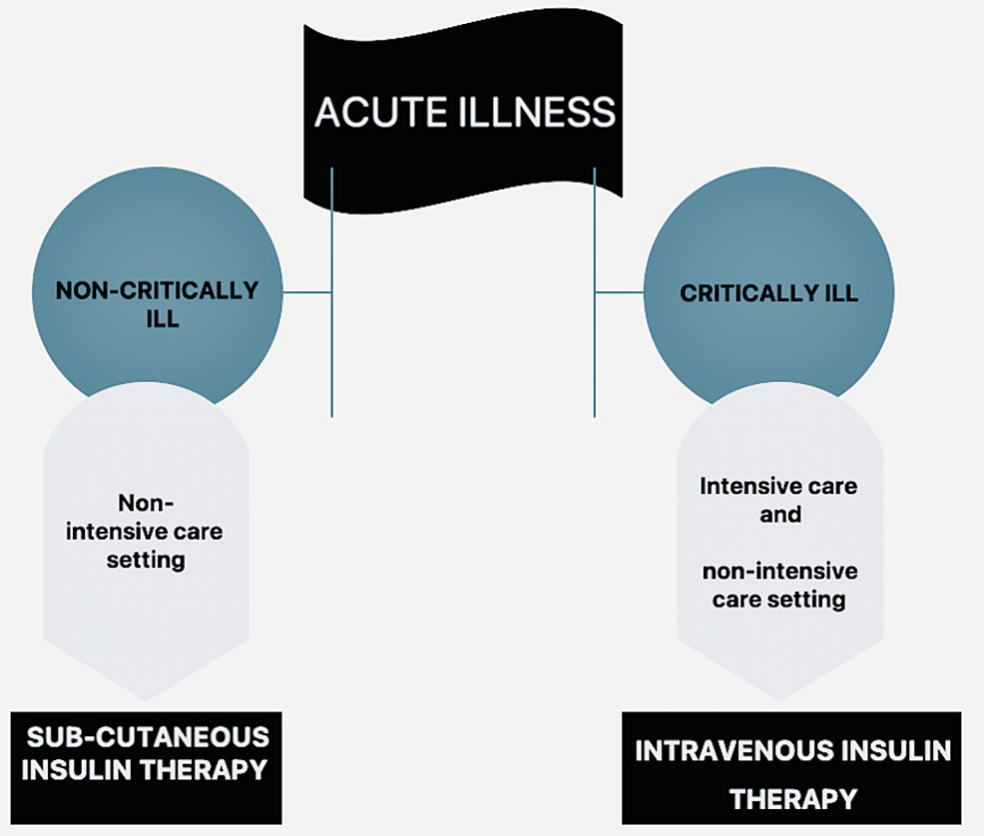
Insulin Administration in the Hospital Image credit: Deepanjali Vedantam

The transition from an ICU setting makes the administration of IV insulin difficult. Hence, shifting to a subcutaneous insulin regimen is recommended [64] (Figure 3). To determine the dose, the American College of Endocrinology recommends using 80% of the 24-hour insulin infusion regimen, with half given as a basal dose and the rest as a bolus [74]. However, critically ill patients not in an ICU and with blood glucose levels above 10 mmol/ L must still be treated with intravenous insulin therapy [75,76].

Non-critically ill hospitalized patients with newly diagnosed hyperglycemia or type 2 diabetes, a subcutaneous basal-bolus insulin regimen in combination with a correction insulin scale is safe to administer according to the 2012 Endocrine Society clinical practice guideline [75] (Table 4). This regimen targets the critical components of insulin requirements:

**Table 4:** Subcutaneous Insulin Administration

Regimen	Application
1. Basal	For fasting state
2. Nutritional	For a post-prandial state
3. Supplemental	For combating unexpected glucose elevations

Novel therapies to manage SIH using glucagon-like peptide-1 (GLP-1) and dipeptidyl-peptidase-4 (DPP-4) inhibitors have been developed [77]. GLP-1 has both insulinotropic and glucagonostatic actions. While given the complicated pathophysiology of SIH, it makes it an extremely effective treatment [78]. Additionally, the mechanism of action is glucose-dependent; therefore, treatment is associated with a very low, if any, risk of hypoglycemia [79]. GLP-1 given Intravenously (1.2 pmol/kg/min) to patients with SIH potentially reduces elevations in blood glucose in response to enteral nutrition [80]. This response is based on the delaying impact of GLP-1 gastric emptying. Still, it can only be observed when the initial gastric emptying was regular, which is not the situation in critically ill patients [81].

In addition to determining the optimal insulin dose, it is critical to consider the nutritional requirements. To prevent hyperglycemia, energy intake must be appropriately handled to avoid excessive glucose consumption and overfeeding. Furthermore, the hormonal environment that promotes excessive gluconeogenesis might also increase protein catabolism [82]. According to research, excess caloric intake may not always reverse this catabolic state. With this in mind, some studies found that providing half of an individual's energy demands would be enough to maintain the same nitrogen balance as "full" feeds while lowering the risk of overt hyperglycemia [82]. While the ideal calorie intake during critical illness is unknown, some temporary underfeeding during an ICU stay may be safe and can assist reduce glucose excursions, especially in a state of severe insulin resistance [83,84]. On a similar issue, good glucose management may help reduce protein catabolism, as preliminary research suggests that moderate glucose control leads to a lower negative nitrogen balance in medical ICU patients [85]. Feeding reduces the hazards of severe hypoglycemia, while a protracted fasting state can exacerbate insulin resistance. Compared with dextrose-containing intravenous solutions, a small amount of enteral nourishment (e.g., 60% of goal rate) has shown to be more successful at significantly reducing the incidence of hypoglycemic episodes in recent small research [86]. A rather large volume (150 mL/hour of 5% dextrose solution) was required to normalize blood sugar levels [86]. Carbohydrates with a lower glycemic index, monounsaturated fatty acids, and fiber may help enhance glycemic control and reduce insulin needs [87,88]. Although earlier trials suggested that glutamine and antioxidant-rich nutritional support may improve survival and glycemic control [89,90], this practice is no longer recommended based on the findings of a recent large multicenter randomized trial. The trial also demonstrated early glutamine administration was associated with worsened mortality.

Discharge plan

Before discharge from the hospital, blood glucose levels must be stabilized. Follow-up and thorough communication with the primary care physician is of utmost importance. All non-diabetic patients who require IIT during critical illness should have an outpatient evaluation in a relaxed state to confirm or rule out diabetes [64]. Screening for diabetes annually is recommended. Furthermore, there may be a compelling argument for routinely offering follow-up testing to patients with blood glucose higher than 11.1 mmol/l aged 30-39, compared to older patients, as the risk of delayed diagnosis may be higher. Screening is not typically offered to individuals under the age of 40, and clinicians may be less likely to diagnose type 2 diabetes due to its relative rarity in this group [91].

Less than one-third of SIH patients retain normal glucose metabolism, and HbA1c tends to gradually deteriorate, providing evidence of the risk of developing pre-diabetes and diabetes following recovery from SIH. Adjustments such as nutritional therapy, exercise, and smoking cessation have been found to delay the progression to pre-diabetes and diabetes [52].

Limitations

This review is based solely on publications found in PubMed. Protocols from studies dating back to 2009 have been cited. We only mentioned data for severely ill adults over the age of 18 and not for populations of younger age groups. A detailed description of the nutritional requirement guidelines has not been covered. Hypoglycemia and its effects on different effects on different organ systems are beyond the scope of this article.

## Conclusions

SIH is a condition that develops in patients undergoing any form of medical stress. An interplay between various cytokines and hormones leads to a state of insulin resistance and increased glucose production. Evidence states that patients suffering from SIH usually have poor outcomes in the hospital. The degree of blood glucose elevations and SHR are used as prognostic indicators. Intravenous insulin infusion is the modality of treatment for the critically ill. Appropriate targets must be set based on the patients’ illness, and routine monitoring has significantly improved outcomes. While increased in-hospital mortality is evident from the studies so far, an increase in morbidity is also seen. The risk of developing type 2 diabetes after recovery is high; hence, a dedicated follow-up by healthcare professionals is necessary. Annual blood work to check HbA1c and blood glucose levels is recommended. Moreover, a target blood glucose range needs to be individualized for each patient in the hospital and at home. However, fixed guidelines to manage SIH are yet to be studied in detail. We have highlighted the guidelines that are widely practiced along with the common obstacles like hypoglycemia which can be overcome by timely measurements of blood glucose and appropriate modifications of the insulin dose. Finally, we feel that the SIH needs more in-depth research studies to construct a more organized and direct approach to diagnosing and managing such a condition.
